# HPV Serology Testing Confirms High HPV Immunisation Coverage in England

**DOI:** 10.1371/journal.pone.0150107

**Published:** 2016-03-09

**Authors:** David Mesher, Elaine Stanford, Joanne White, Jamie Findlow, Rosalind Warrington, Sukamal Das, Richard Pebody, Ray Borrow, Kate Soldan

**Affiliations:** 1 HIV & STI Department, Centre for Infectious Disease Surveillance and Control, Public Health England, London, United Kingdom; 2 Vaccine Evaluation Unit, Public Health England, Manchester Royal Infirmary, Manchester, United Kingdom; 3 Immunisation, Hepatitis and Blood Safety Department, Centre for Infectious Disease Surveillance and Control, Public Health England, London, United Kingdom; 4 Respiratory Diseases Department, Centre for Infectious Disease Surveillance and Control, Public Health England, London, United Kingdom; University of Cincinnati College of Medicine, UNITED STATES

## Abstract

**Background:**

Reported human papillomavirus (HPV) vaccination coverage in England is high, particularly in girls offered routine immunisation at age 12 years. Serological surveillance can be used to validate reported coverage and explore variations within it and changes in serological markers over time.

**Methods:**

Residual serum specimens collected from females aged 15–19 years in 2010–2011 were tested for anti-HPV16 and HPV18 IgG by ELISA. Based on these results, females were classified as follows: seronegative, probable natural infection, probable vaccine-induced seropositivity, or possible natural infection/possible vaccine-induced seropositivity. The proportion of females with vaccine-induced seropositivity was compared to the reported vaccination coverage.

**Results:**

Of 2146 specimens tested, 1380 (64%) were seropositive for both types HPV16 and HPV18 and 159 (7.4%) positive for only one HPV type. The IgG concentrations were far higher for those positive for both HPV types than those positive for only one HPV type. 1320 (62%) females were considered to have probable vaccine-induced seropositivity. Among vaccine-induced seropositives, antibody concentrations declined with increasing age at vaccination and increasing time since vaccination.

**Conclusions:**

The proportion of females with vaccine-induced seropositivity was closest to the reported 3-dose coverage in those offered the vaccination at younger ages, with a greater discrepancy in the older females. This suggests either some under-reporting of immunisations of older females and/or that partial vaccination (i.e. one- or two-doses) has provided high antibody responses in 13–17 year olds.

## Introduction

A national HPV immunisation programme was introduced throughout the UK in September 2008 with routine vaccination offered to all girls aged 12–13 years and a catch-up programme in the first two years offering the vaccination to all girls up to the age of 18 years. From 2008 to 2011, the bivalent vaccine was offered with a change in September 2012 to the quadrivalent vaccine. HPV vaccination is offered free of charge to all girls. The immunisation programme is primarily delivered in schools but also in General Practitioners (GPs) and other health care services, particularly for the older catch-up cohorts. Reported vaccination coverage has been high with over 80% of girls in the routine cohorts completing the three dose schedule [1–4]. Reported coverage is based on data provided by local areas, collated and monitored by Public Health England (PHE).

In females, following a natural infection with HPV, a detectable antibody response is only detected around 50–70% of the time [5–8] and this response is usually fairly weak. Vaccination induces seroconversion in close to 100% of recipients and results in substantially higher average IgG concentrations than following natural infection [9].

Accurate, validated knowledge of HPV vaccination coverage is important to assess the likely direct impact of the HPV immunisation programme as well as the potential indirect effect of herd protection among the unvaccinated. Monitoring of serological markers can also enable vigilance for potential lower levels of direct protection from the immunisation programme within certain sub-groups, and for changes in immunogenicity over time, i.e. antibody waning, which may presage reductions in protection.

We have used distributions of anti-HPV 16 and HPV 18 IgG concentrations to classify sera from a sample of young females in England as probable vaccine-induced seropositive or probable natural infection. We compare the resulting estimates of coverage derived from anti-HPV IgG concentrations to reported vaccination coverage, and explore associations between antibody levels and age at vaccination and time since vaccination.

## Materials and Methods

### Ethics statement

National Research Ethics Service (NRES) approval for the sero-epidemiological surveillance of the National Immunisation programme of England and Wales (Research Ethics Committee number 05/Q0505/45) was granted by the Joint University College London/University College London Hospital (UCL/UCLH) Committees on the Ethics of Human Research.

Patient consent was not required as this study made use of anonymised specimens (with no patient identifiable data) which were collected and tested as part of Public Health Surveillance conducted to monitor the HPV vaccination programme.

### Residual serum specimens

**Fig 1:**
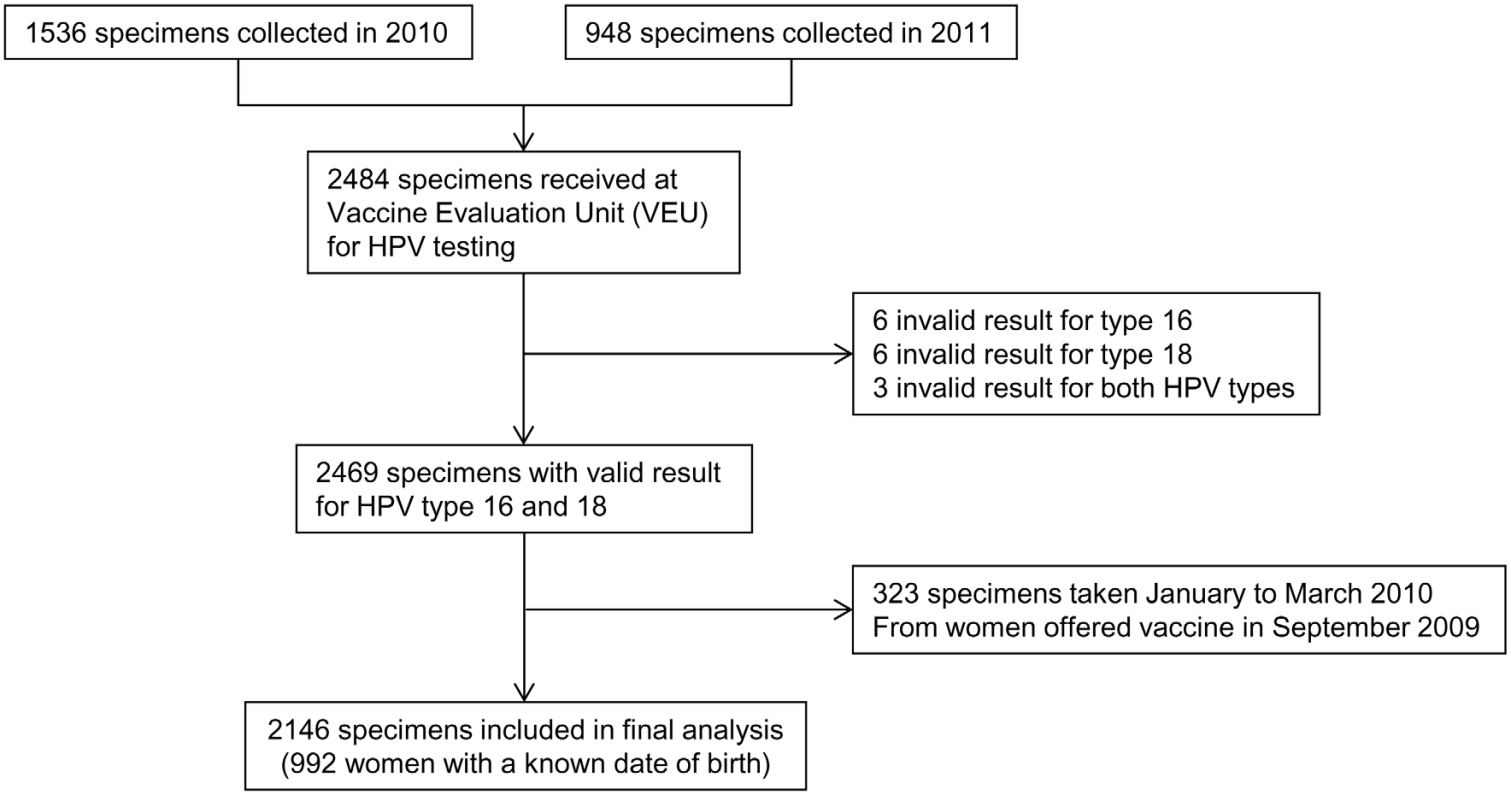
Flow chart of eligible samples.

Serum specimens from females aged 15–19 years were obtained from the PHE Seroepidemiology Unit (SEU). The SEU routinely collects residual serum specimens after diagnostic microbiological tests for seroepidemiological studies of infections of public health importance for which vaccines are available or under development. Contributing laboratories in England provide anonymised specimens with age at collection, sex, and date of collection. Sera from immunocompromised individuals and repeat sera from the same individuals are excluded [10]. Where possible, laboratories identify specimens that originated from Genitourinary Medicine (GUM) clinics. We increased collection of specimens from females aged 15–19 years old (i.e. who would have been eligible to receive the bivalent HPV vaccine as part of the national HPV immunisation programme) by approximately 1000 specimens per year for the purposes of this study. A total of 2484 serum specimens were collected from 12 contributing laboratories between January 2010 and December 2011 (Fig 1). Where exact age at sample collection was available, this was used to generate the age and calendar year that HPV vaccination would have been offered: this was available for 992/2146 (46.2%) of women. For the remainder, with age in years available, likely year of eligibility for HPV vaccination was estimated. Specimens collected in January-March following the due date of first vaccine dose were excluded in order to study seroprevalence after, not during, the scheduled full course of immunisation. Analyses considering time since vaccination and age at vaccination were restricted to women with a known exact age.

### HPV testing and serological coverage

Specimens were tested at the PHE Vaccine Evaluation Unit (VEU), Manchester for IgG to HPV types 16 and 18 using a type-specific ELISA and all assay critical reagents, including Virus Like Particles (VLPs), transferred from GlaxoSmithKline [11]. Briefly, VLP16 and VLP18 antigens, purified from recombinant Baculovirus were pre coated onto separate 96 well microtitre plates for between 60 and 120 hours at 4°C. Following blocking to prevent non-specific binding, test, negative control, positive control and standard serum were added to VLP 16 and VLP 18 plates, in serial two-fold dilutions and incubated for 60 minutes at room temperature. Specific bound antibody was detected using horseradish peroxidase goat anti-human IgG conjugate and developed with a specific chromogenic substrate. Optical density was determined at 450nm with a 620nm reference. Quantitative results were calculated from the standard and expressed in arbitrary ELISA units per millilitre (EU/mL). The lower limit of quantitation of the assay at the VEU was 19 and 18 EU/mL for HPV16 and HPV18, respectively, with values below this classed as seronegative.

**Fig 2:**
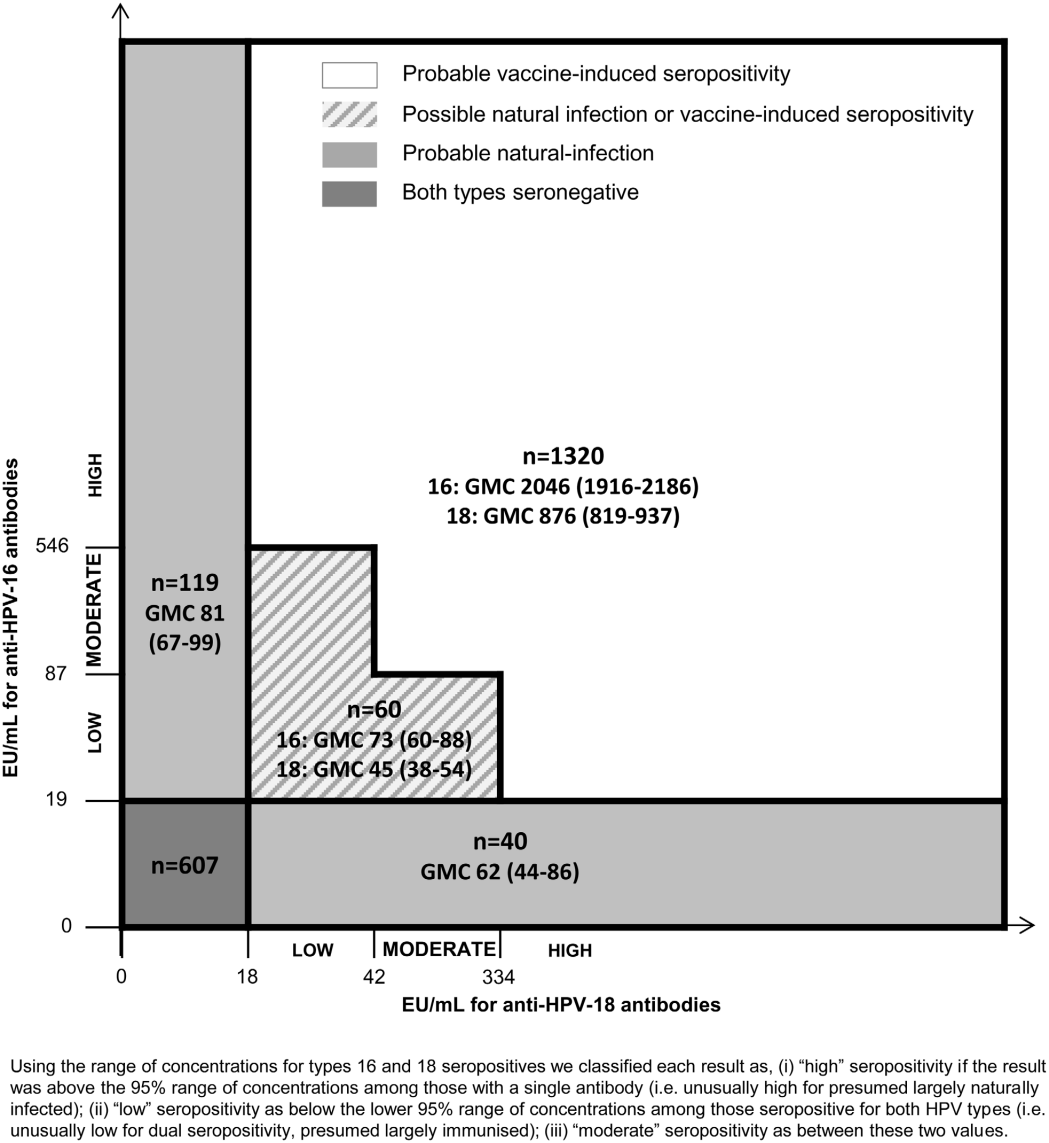
Definition of natural infection and vaccine-induced seropositivity. Abbreviations: EU/mL = ELISA units per millilitre per mililitre; GMC = Geometric mean concentration.

Antibody concentrations are presented as geometric mean concentrations (GMCs) among seropositive specimens. Whilst average antibody levels following vaccination are far higher than those following natural infection, the ranges overlap. Using the range of concentrations for types 16 and 18 seropositives we classified each result as, (i) “high” seropositivity if the result was above the 95% range of concentrations among those with a single antibody (i.e. unusually high for presumed largely naturally infected); (ii) “low” seropositivity as below the lower 95% range of concentrations among those seropositive for both HPV types (i.e. unusually low for dual seropositivity, presumed largely immunised); (iii) “moderate” seropositivity as between these two values. Using this grading, we then classified “probable” vaccine-induced seropositivity as seropositive for both types with high concentration for at least one type or moderate concentrations for both types and “probable” natural infection as seropositive for one type only. Specimens with low seropositivity for both types or low seropositivity for one type and moderate for the other were classified as “possible” natural infection or vaccine-induced seropositivity (Fig 2). Serological coverage estimates were calculated as vaccine-induced seropositives divided by the total number of sera with valid test results.

### Reported HPV vaccination coverage

Data on reported coverage for each birth cohort included in our seroprevalence data were obtained from published tables [1]. Briefly, annual data on the number of girls receiving at least one, at least two doses or all three doses of the vaccine for each area are submitted to the ImmForm website, a web-based reporting system managed by PHE, using denominators based on the appropriate age-specific school-roll data for females, obtained from the Department for Education.

For comparison with serological coverage, vaccination coverage was estimated using the published coverage for the 10 geographical areas (Strategic Health Authorities) of the laboratories submitting serum specimens, as well as using national level data. Published coverage, reported by academic year (September to August), was used to estimate coverage by age and calendar year. As estimates using Strategic Health Authority data and national data for reported vaccination coverage were similar, the estimates from national data were used.

## Results

A valid result for both HPV 16 antibodies and HPV 18 antibodies was available for 2469 of the 2484 specimens (99.4%). 323 specimens with date of collection during January to March of the year following the due date of first vaccine dose were excluded: 2146 specimens were included in the analysis (Fig 1).

The mean age of females providing a specimen was 17.9 years (SD 1.4 years). Around one-third of specimens were known to have originated from GUM clinics (ranging from 0% to 93% by contributing laboratory). The mean age was similar for specimens from GUM clinics and those from unspecified source clinics (17.8 years and 17.9 years, respectively).

### Seropositivity for vaccine HPV-types

Across all ages, 64% (1380) of specimens were seropositive for both HPV 16 and HPV 18. Seropositivity for HPV 16 only and for HPV 18 only was found in 5.5% (119) and 1.9% (40) of specimens, respectively. 28% (n = 607) were seronegative for both HPV types (Fig 2). The GMCs were over 10-fold higher for specimens which were seropositive for both HPV types (GMC of 1770 EU/mL for HPV type 16 and 770 EU/mL for HPV type 18) than among those seropositive for only one type (GMC of 81 EU/mL for HPV type 16 and 62 EU/mL for HPV type 18).

Overall seropositivity for HPV16 and/or HPV18 was higher in specimens known from GUM clinics (76.3% vs. 69.0% for HPV 16 and/or 18) and specimens from younger ages (Table 1).

**Table 1:** Seropositivity for HPV16 and 18 by clinical setting and laboratory sending specimens

	Number with valid result	Proportion seropositive for HPV 16 and/or 18	Vaccine-induced seropositivity	Natural infection seropositivity
	n	n (%)	n (%)	n (%)
**Total**	*2*,*146*	*1*,*539 (71*.*7)*	*1*,*320 (61*.*5)*	*219 (10*.*2)*
**Clinical setting**				
Genito-urinary Medicine (GUM) clinic	798	609 (76.3)	535 (67.0)	74 (9.3)
Unknown clinic setting	1,348	930 (69.0)	785 (58.2)	145 (10.8)
**Age specimen taken**				
15 years	314	249 (79.3)	236 (75.2)	13 (4.1)
16 years	361	286 (79.2)	263 (72.9)	23 (6.4)
17 years	324	236 (72.8)	200 (61.7)	36 (11.1)
18 years	523	364 (69.6)	303 (57.9)	61 (11.7)
19 years	624	404 (64.7)	318 (51.0)	86 (13.8)
**Laboratory**^a^				
North East				
Newcastle	290	240 (82.8)	207 (71.4)	33 (11.4)
North West				
Manchester	289	209 (72.3)	183 (63.3)	26 (9.0)
Yorkshire and The Humber				
Leeds	530	378 (71.3)	314 (59.2)	64 (12.1)
East Midlands				
Cambridge	95	61 (64.2)	53 (55.8)	8 (8.4)
Leicester	172	145 (84.3)	138 (80.2)	7 (4.1)
West Midlands				
Birmingham	27	12 (44.4)	9 (33.3)	3 (11.1)
London				
Barts and The London	199	112 (56.3)	93 (46.7)	19 (9.5)
St George’s Hospital	105	50 (47.6)	37 (35.2)	13 (12.4)
South Central				
Southampton	9	6 (66.7)	5 (55.6)	1 (11.1)
South East				
Brighton	49	30 (61.2)	24 (49.0)	6 (12.2)
South West				
Bristol	25	22 (88.0)	19 (76.0)	3 (12.0)
Exeter	331	257 (77.6)	222 (67.1)	35 (10.6)
Gloucester	25	17 (68.0)	16 (64.0)	1 (4.0)

^a^ p-value for heterogeneity across laboratories; p<0.0001 for proportion seropositive for HPV 16 and/or 18, p<0.0001 for proportion with vaccine-induced seropositivity, p = 0.415 for proportion with natural infection seropositivity.

### Vaccine-induced seropositivity (VIS)

**Fig 3:**
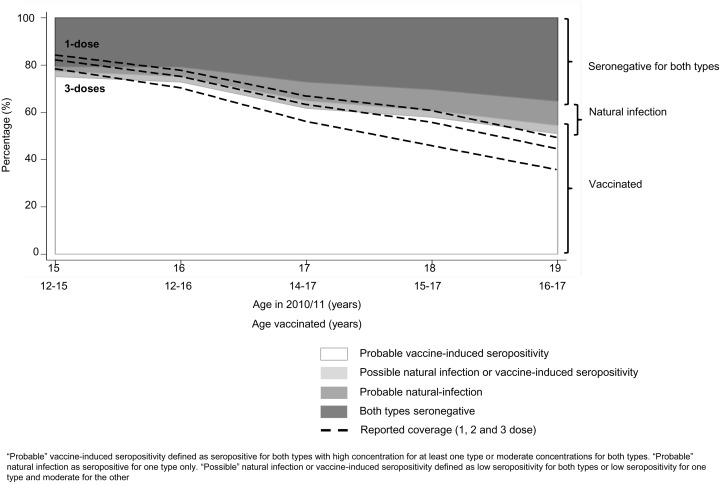
Published HPV vaccine coverage and vaccine induced seropositivity by age (n = 2,146).

Within this sample of serum, using the methods described, probable vaccine-induced seropositivity (VIS) was defined as sera with antibody concentrations above 546 EU/mL for HPV 16 or above 334 EU/mL for HPV 18 (and seropositive for HPV 18 and HPV 16, respectively), or above 87 EU/mL for HPV 16 and above 42 EU/mL for HPV 18 (Fig 2). The overall proportion of females with probable VIS was 61.5% (1320) with GMCs of 2046 EU/mL (95%CI 1916–2186) and 876 EU/mL (95%CI 819–937) for HPV 16 and 18 respectively. An additional 2.8% (60) were possible natural infection or possible VIS (with GMCs of 72.9 EU/mL (95%CI 60.1–88.5) for HPV 16 and 45.1 EU/mL (95%CI 37.8–53.8) for HPV 18). The proportion of females with vaccine-induced seropositivity was slightly lower than the reported three-dose coverage for 15 year olds but higher at older ages. There was increasing discrepancy between reported coverage and the proportion of females with vaccine-induced seropositivity with increasing age (Table 2 and Fig 3).

**Table 2:** Seropositivity for HPV16 and 18 amongst all specimens tested for both HPV types, by age.

	Age in years					
HPV type	15	16	17	18	19	*Total*
**All women**	***314***	***361***	***324***	***523***	***624***	***2*,*146***
Both types negative	21% (65)	21% (75)	27% (88)	30% (159)	35% (220)	*28% (607)*
**Natural infection seropositivity:**						
- Probable 18 only	1.0% (3)	0.8% (3)	1.9% (6)	2.1% (11)	2.7% (17)	*1*.*9% (40)*
- Probable 16 only	1.0% (3)	3.3% (12)	6.2% (20)	6.9% (36)	7.7% (48)	*5*.*5% (119)*
- Probable 16 or 18	1.9% (6)	4.2% (15)	8.0% (26)	9.0% (47)	10.4% (65)	*7*.*4% (159)*
- Probable and possible	4.1% (13)	6.4% (23)	11.1% (36)	11.7% (61)	13.8% (86)	*10*.*2% (219)*
**Vaccine-induced seropositivity:**						
- Probable	75% (236)	73% (263)	62% (200)	58% (303)	51% (318)	*62% (1320)*
- Probable and possible	77% (243)	75% (271)	65% (210)	61% (317)	54% (339)	*64% (1380)*
Expected 1-dose (national)	84.2%	77.7%	66.7%	60.5%	48.9%	-
Expected 2-dose (national)	82.1%	75.0%	63.1%	55.5%	44.1%	-
Expected-3-dose (national)	78.3%	70.1%	55.8%	45.5%	35.1%	-

“Probable” vaccine-induced seropositivity defined as seropositive for both types with high concentration for at least one type or moderate concentrations for both types. “Probable” natural infection as seropositive for one type only. “Possible” natural infection or vaccine-induced seropositivity defined as low seropositivity for both types or low seropositivity for one type and moderate for the other.

**Fig 4:**
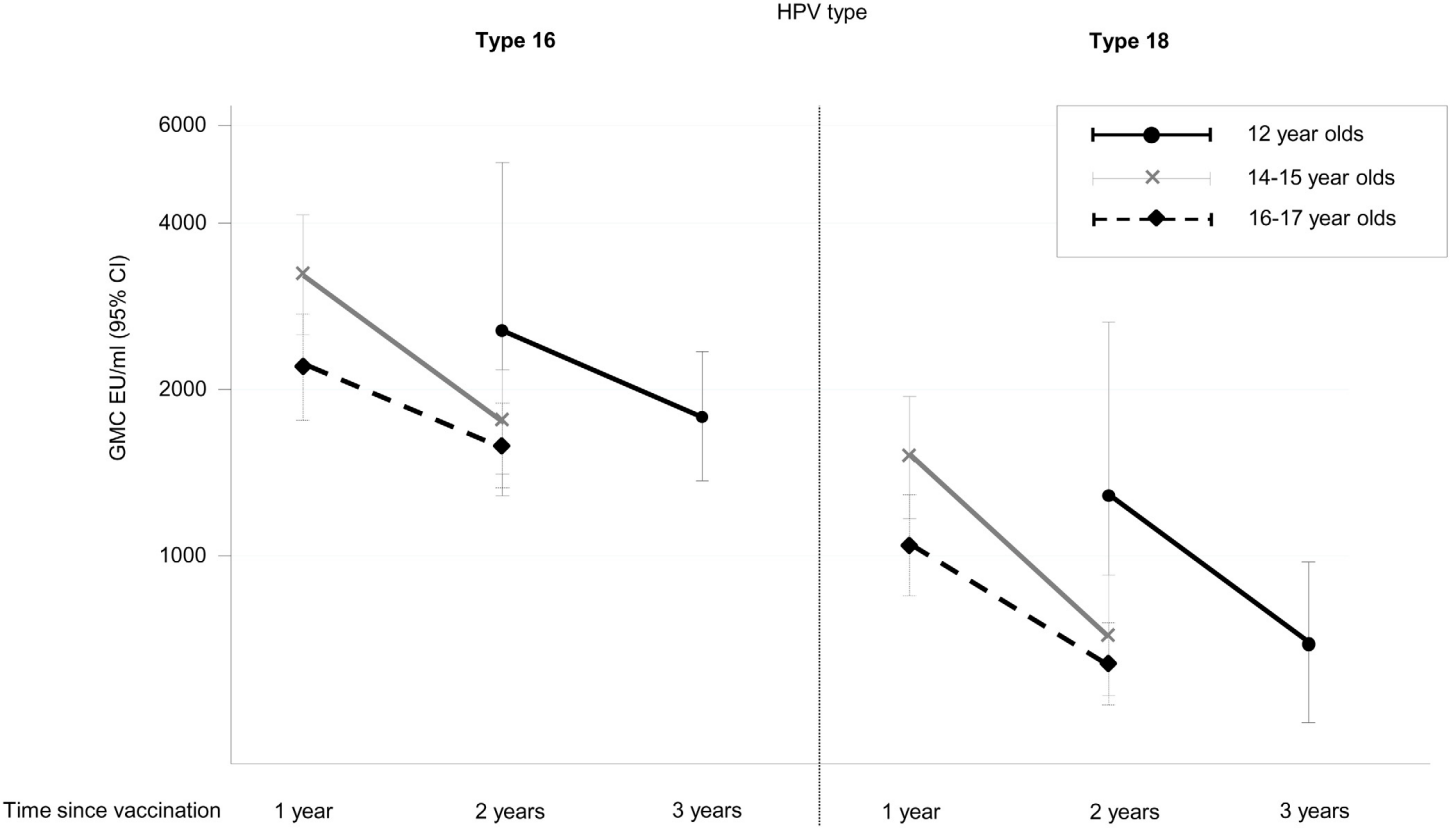
Geometric mean concentrations (GMCs) for HPV type 16 and 18 among those with probable vaccine-induced seropositivity. Stratified by age at HPV vaccination and time since vaccination. Restricted to women with a known date of birth (n = 564). GMC for probable natural infection: 71 EU/mL for type 16; 36 EU/mL for type 18. Time from 1^st^ September in year first offered the HPV vaccine as part of the national immunisation programme to the date serology specimen was taken. Abbreviations: EU/mL = ELISA units per millilitre per millilitre

Among the probably VIS, GMCs for HPV16 were higher than GMCs for HPV18 at all ages. For both HPV 16 and HPV 18, GMCs declined with increasing time since vaccination (up to 3-years data available). Specimens estimated to have been taken at equal times after vaccination tended to have higher GMCs if vaccinated at younger ages (at two-years following vaccination, GMCs in 12 year olds were 2561 EU/mL (95%CI 1273–5154) and 1296 EU/mL (95%CI 632–2656) for types HPV16 and HPV18 respectively, whereas in 14–17 year olds these were lower at 1631 EU/mL (95%CI 1422–1871) and 669 EU/mL (95%CI 581–770) respectively) (Fig 4).

### Natural infection seropositivity

The GMCs for those with probable natural infection (i.e. seropositive for only one HPV type) were 81.1 EU/mL (95%CI 66.6–98.7) for HPV 16 and 61.7 EU/mL (95%CI 44.2–86.0) for HPV 18. GMCs for those seropositive for both types but with possible natural infection were similar (72.9 EU/mL (60.1–88.5) and 45.1 EU/mL (37.8–53.8) for HPV 16 and 18, respectively). The proportion of females with probable or possible natural infection was similar in specimens known to be submitted from GUM clinics (9.3% vs. 10.8% for those from a GUM clinics vs. those from an unspecified clinic, respectively). The probable and possible natural infections increased with increasing age (Fig 3).

## Discussion

Serological surveillance confirms high coverage of the HPV vaccination programme in young females in England, particularly in those offered the vaccine at school age. The higher proportion with vaccine-induced seropositivity compared to reported three-dose coverage, particularly evident in the older females (offered HPV vaccination at an older age), suggests that three-dose coverage in the catch-up cohorts could be higher than reported, or that two-dose coverage at these ages is associated with high antibody responses, or both.

We used the results from serological testing to determine the vaccination status of females. Previous studies have shown only a relatively small proportion of females have natural seropositivity for both HPV types 16 and 18 [12]. Conversely, data from clinical trials show close to 100% of vaccinated females seroconvert for both HPV types [13] with no substantial waning of seropositivity up to seven years following vaccination [14]. Whilst antibody levels are generally far higher in vaccinated females [14], the antibody levels required to protect against HPV infection are unknown and there is an overlap in the ranges of concentrations in vaccinated and unvaccinated females which could have led to some limited misclassification. Our data are consistent with clinical trial data in showing a 10 to 20-fold higher GMC in those with dual seropositivity (presumed largely vaccinated) than those seropositive for only one HPV type (presumed largely natural infections). Those classified as “possible natural infection or vaccine-induced seropositivity” likely reflect the group with natural infection for both HPV types although this proportion is slightly higher than that detected by competitive Luminex assay (cLIA) in a pre-immunisation survey using the same serum collection although a different assay (2.8% in this study compared to 1.8% in 15–19 year old females included in the survey performed prior to the introduction of the HPV immunisation programme [12]).

The average antibody concentrations declined with both increasing age at vaccination and increasing time since vaccination (Fig 4). This is consistent with other observations of immunogenicity by age and the fact that highest levels are reported immediately after HPV vaccination with a slight decrease subsequently [14;15]. Partial vaccination (one or two doses only) was also reportedly more common at older ages of vaccination. We present data up to three years post-vaccination which demonstrate that those with probable vaccine-induced seropositivity still had far greater antibody levels than those following a natural infection. Longer-term serosurveillance, and infection surveillance, is needed to monitor the significance of waning antibody concentrations.

Quantitative antibody concentrations from the study are not comparable to those from studies which use different assays/cut-offs, because currently no international HPV standard reference serum exists, and different laboratories therefore use ‘in-house’ standard sera making direct comparisons nonviable. One point for consideration is that the ELISA methodology we applied used an increased lower limit of quantification compared to previous reports (we use a lower limit of 19 EU/mL for HPV16 and 18 EU/mL for HPV18 whereas 8 EU/mL and 7 EU/mL respectively have been used previously) [11]. Using this higher cut-off, 92 women previously considered HPV seropositive were reclassified as seronegative. Although this change resulted in a modest decrease in sensitivity of the ELISA, this had little effect on the classification of specimens as those with natural infection seropositivity or vaccine-induced seropositivity.

Residual serum specimens for this surveillance are taken from females attending for diagnostic and screening tests, hence may not be representative of the general population. The reason for the initial test where the serum sample was taken is not provided to SEU along with the sample. Where it was known that a sample originated from a GUM clinic, this was indicated. Analyses were performed separately for known GUM samples and other samples and results were very similar (data not shown). No data are collected on social deprivation, ethnicity or country of birth of females although since England has free access to health care this reduces the potential bias associated with health-seeking behaviour. One previous paper suggested that the comparable results between coverage data and other vaccine seroconversion rates provide some assurance of the representativeness of these specimens [10].

Vaccination coverage may have been slightly under-reported for the older catch-up vaccination cohorts. In this group, vaccination was largely performed outside of schools and revised estimates of coverage to include data on immunisations given late in ‘mop-up’ sessions were not readily available in all areas hence the quality of these data was less certain (2). This would be consistent with the greater differences between reported coverage and vaccine-induced seropositivity observed in females offered vaccination at older ages. However, we also consider that the proportion reported to have received only one or two doses of the vaccine is far greater in older ages (just under 5% in those vaccination in the routine cohorts compared to around 15% in the older catch-up cohorts). The majority of females who had received two-doses would have received the first two doses of the vaccine (i.e. with just one or two months between doses). Limited data are available on antibody responses following such a schedule. Data from trials have shown 100% of females seroconvert after two-doses given six months apart with non-inferior antibody concentrations compared to females receiving three-doses [16]. We were therefore unable to distinguish differences in antibody concentrations in females receiving one- or two-doses of the vaccine from females receiving all three doses. Therefore the higher than expected level of vaccine associated seroprevalence in the older cohorts could suggest that three-dose coverage in the catch-up cohorts have been higher than recorded and/or that vaccination of 13–17 year olds with one or two-doses of the vaccine generated high antibody concentrations.

Future studies using these methods will explore serological coverage in certain demographic and behavioural subgroups to identify those relatively lacking in direct vaccine-protection and will monitor antibody levels over longer times since vaccination. This will be an important part of assessing longer term protection.

## Supporting Information

S1 Data Minimal data set. Field names: gender; yearofcollection: Year sample taken; age_year: Age at sample date; result_16: HPV16 antibody concentration (EU/mL); result_18: HPV18 antibody concentration (EU/mL).
(XLSX)
